# Fall Detection by Deep Learning-Based Bimodal Movement and Pose Sensing with Late Fusion

**DOI:** 10.3390/s25196035

**Published:** 2025-10-01

**Authors:** Haythem Rehouma, Mounir Boukadoum

**Affiliations:** Département d’informatique, Université du Québec à Montréal, Montréal, QC H2X 3Y7, Canada

**Keywords:** fall detection, multimodal learning, LSTM autoencoder, Transformer, IMU, pose estimation, elderly care, late fusion, nighttime monitoring

## Abstract

The timely detection of falls among the elderly remains challenging. Single modality sensing approaches using inertial measurement units (IMUs) or vision-based monitoring systems frequently exhibit high false positives and compromised accuracy under suboptimal operating conditions. We propose a novel bimodal deep learning-based bimodal sensing framework to address the problem, by leveraging a memory-based autoencoder neural network for inertial abnormality detection and an attention-based neural network for visual pose assessment, with late fusion at the decision level. Our experimental evaluation with a custom dataset of simulated falls and routine activities, captured with waist-mounted IMUs and RGB cameras under dim lighting, shows significant performance improvement by the described bimodal late-fusion system, with an F1-score of 97.3% and, most notably, a false-positive rate of 3.6% significantly lower than the 11.3% and 8.9% with IMU-only and vision-only baselines, respectively. These results confirm the robustness of the described fall detection approach and validate its applicability to real-time fall detection under different light settings, including nighttime conditions.

## 1. Introduction

Falls by the elderly remain a major public health concern due to their effect on autonomy, quality of life, and mortality [1,2], and their timely and reliable detection is essential, particularly for older adults living independently. However, distinguishing actual falls from daily activities remains challenging under realistic conditions, especially at night when visual cues are degraded by low illumination and occlusions from blankets or furniture [3,4,5]. The current fall detection systems rely primarily on single modality sensing using inertial measurement units (IMUs) or vision-based monitoring [1,2,3]. IMU-based methods, often employing simple thresholds or shallow classifiers [6,7,8,9,10], are inexpensive and minimally intrusive, but they lack spatial contextualization and frequently generate false alarms. Vision-based methods provide richer contextual information through background subtraction, pose estimation, and deep classifiers [11,12,13,14]. Yet, the performance of vision-based systems can degrade sharply under nocturnal conditions [5].

To obtain more accurate and reliable fall detection, multimodal sensing systems that combine information from different sensors have been increasingly explored, notably by combining inertial and vision sensors [3,15]. The sensor fusion can be performed early by combining the individual sensor outputs before further processing, with the risk of increasing noise in the processing chain, or late by having each output processed independently, and the results merged before the final decision, enabling better calibration, fault isolation, and resilience against modality-specific failures [16,17]. In this regard, decision-level fusion is more robust than early fusion, while they both endow multimodal systems with the potential of better detection performance than single modality solutions.

In this study, we propose a deep learning-based bimodal framework for fall detection that can operate at night, thanks to the integration of inertial abnormality detection by an unsupervised LSTM autoencoder [18] and pose assessment by a Transformer-based vision module [19]. By adopting decision-level fusion, the complementary strengths of IMU and vision sensing are leveraged by having the inertial cues ensure robustness when vision is impaired, and the visual cues provide spatial verification when inertial data is ambiguous.

A cohort of 16 participants was used to validate the framework, as training experiments showed the performance gains to reach a plateau at ≈12 participants as will be shown. Personalization experiments using few-shot learning also showed that at least 95% performance could be recovered with only five annotated sequences per new subject, thus mitigating inter-individual variability and addressing practical deployment needs.

Finally, RGB cameras were used for vision sensing thanks to their low cost, broad availability, and acceptability in private environments as used by our framework, since it operates by transforming the raw video frames into abstract skeletal keypoints, hence thwarting subject identification while preserving context for fall detection. Moreover, our preliminary experiments confirmed that robust performance (F1 > 96%) could be maintained with less than 5 lux illumination, thus mitigating the need for specialized sensors.

Our main contributions are as follows: (1) a decision level fused IMU–RGB architecture for nocturnal operation, (2) the combination of an unsupervised LSTM autoencoder for inertial abnormality detection and a Transformer-based vision module modeling spatiotemporal pose from 2D skeletal landmarks, (3) a few-shot personalization protocol for rapid user adaptation, (4) a systematic comparison showing the superiority of the proposed approach over unimodal and early-fusion baselines in accuracy and false alarms.

## 2. Related Work

### 2.1. Assisted Living Technologies for Fall Detection

Fall detection systems are commonly grouped into three generations [1]. The first generation relied on user-triggered alarms (e.g., wearable panic buttons) that could fail when the user is incapacitated. The second-generation introduced wearable IMUs with thresholding and classical machine learning (ML) classifiers. Today, the third-generation systems seek to combine artificial intelligence and multimodal sensing, leveraging deep neural networks to improve the detection process and context understanding.

### 2.2. IMU-Based Fall Detection

IMU-only approaches remain attractive for cost and privacy reasons [3,4], and they range from simple threshold-based systems [4,20] to classical ML-based classifiers such as SVM, random forests, decision trees, and *k*-NN [6,21,22,23,24], and deep learning models recently. For example, Zhang et al. [25] developed a dual-stream convolutional neural network with a self-attention mechanism that learns discriminative features from accelerometer and gyroscope data and assigns weights to different phases of the fall signal. The model outperformed traditional threshold-based and shallow learning approaches on public datasets, demonstrating that neural networks can enhance fall detection accuracy while remaining embeddable. However, while effective under controlled conditions, they often fail to disambiguate daily fall-like activities because of the lack of spatial confirmation, thus leading to a high false alarm rate [3]. More recent deep learning models such as unsupervised LSTM autoencoders can improve the detection robustness by sensing deviations from learned normal motion [18], but they also suffer from the lack of spatial verification.

### 2.3. Vision-Based Fall Detection

Vision-based methods offer a richer spatial context via silhouettes, skeletal landmarks, thermal or depth sensing, and classification thereof [5,10,11,26,27,28]. However, their performance is constrained in realistic settings such as low light and occlusions at night, and privacy concerns can limit their acceptance in homes [5,12].

### 2.4. Multimodal Approaches and Fusion Techniques

IMU and vision integration have been widely explored for potentially benefiting from complementary strengths [29,30], but the process is not trivial. As mentioned, early fusion using feature-level concatenation [31,32,33] brings the risk of propagating modality-specific noise and redundancy, thus reducing generalization potential [16,32]. Decision-level (late) fusion fares better in this respect, by exploiting the two modalities independently and combining their predictions at the end. Prior works combining deep outputs or video–accelerometer cues reported improved robustness, though not focusing on nocturnal operation [32,34].

### 2.5. Remaining Gaps and Proposed Contributions

The persisting gaps in the state of the art include (i) limited evaluation in nocturnal low-light conditions typical of bedrooms [5,12]; (ii) persistent false positives undermining trust [35]; (iii) lack of systematic multimodal integration with abnormality detection (e.g., LSTM autoencoders) [18]; and (iv) limited use of attention-based temporal modeling within multimodal pipelines [8,19]. This work addresses these gaps by introducing a bimodal IMU–RGB framework with decision-level fusion that couples an unsupervised LSTM autoencoder for inertial abnormality detection [18] with a Transformer-based vision module for pose-sequence analysis [19,36].

## 3. Proposed Bimodal Decision-Level Fusion Architecture

The proposed bimodal fall detection architecture consists of two distinct processing streams: (i) a vision-based stream exploiting video-based skeletal landmarks and pose evolution, and (ii) an inertial-based stream using an LSTM autoencoder for inertial abnormality detection. Both streams operate independently, and their outputs are fused by a decision-level rule for robust and reliable fall detection.

### 3.1. Video Processing Pipeline and Transformer-Based Fall Detection

The vision processing module begins by using MediaPipe Blazepose from Google Research [37,38] to extract 2D skeletal landmarks from the RGB video frames. BlazePose uses a detector-tracker machine leaning architecture specifically designed for real-time operation under challenging conditions, including moderate occlusions, blanket coverage and poor illumination. The two-step pose estimation pipeline proceeds as follows: (1) a lightweight detector locates a region-of-Interest (ROI) around the upper body, predicting virtual keypoints to ensure a normalized and rotation-invariant body pose region; (2) a depthwise-separable convolutional neural network regresses 33 anatomical landmark coordinates and assigns a visibility score between 0 (not visible) and 1 (fully visible) to each one of them.

At each video frame at time t, the current landmarks serve to update the ROI for the frame at t+1, to allow continuous tracking without frequent full-frame detections. This tightly coupled detector–landmark interaction guarantees robust landmark estimation even under adverse conditions, including partial occlusions, low-light, and typical nighttime visual perturbations.

In this study, 2D skeletal landmarks were adopted instead of 3D, because 2D inference better satisfies the CPU-only real-time budget of the target platform and can be more robust under nocturnal low-light conditions where 3D depth-based estimation frequently fails. Furthermore, modern 2D keypoint extractors provide stable tracking and sufficient postural cues for the downstream Transformer. Future work will investigate 3D variants once illumination and computing constraints can be relaxed.

**Figure 1:**
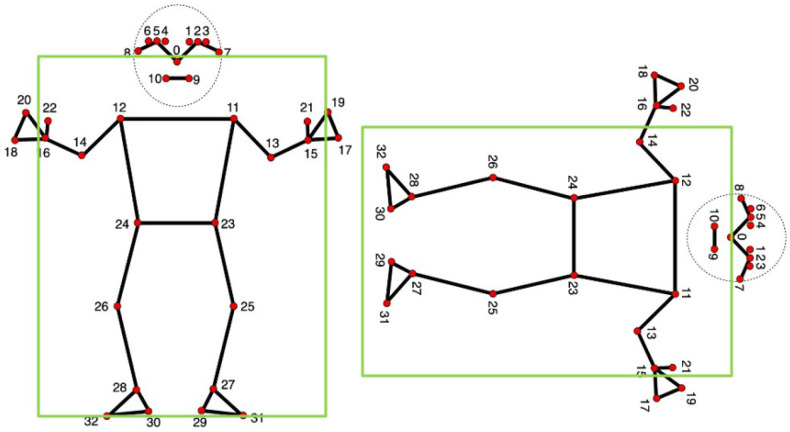
Skeletal MediaPipe model [42] with bounding box, showing the 33 anatomical landmark points for pose estimation: Standing up (**left**), lying down (**right**). The bounding box’s aspect ratio helps detect falls.

Figure 1 illustrates upright vs. prone configurations from BlazePose. The evolution of their bounding box’s aspect ratio can be used to provide a coarse postural cue.

Following landmark extraction, the obtained coordinates in normalized units are converted to pixel coordinates by

(1) $$x_{i}^{pixel}=x_{i}^{norm}.w_{\mathrm{frame}}\;\mathrm{and}\;y_{i}^{pixel}=y_{i}^{norm}.h_{\mathrm{frame}}$$

where $w_{\mathrm{frame}},\;h_{\mathrm{frame}}$ denote the frame dimensions in pixels. Then, a coarse bounding box is computed for posture estimation, with its extreme coordinates derived from the subset of the landmark points with visibility greater than a threshold (0.50 in this work):

(2) $$x_{\min}=\underset{i}{\min}\;x_{i}^{pixel},\;x_{\max}=\underset{i}{\max}\;x_{i}^{pixel}$$

(3) $$y_{\min}=\underset{i}{\min}\;y_{i}^{pixel},\;y_{\max}=\underset{i}{\max}\;y_{i}^{pixel}$$

Given the previous coordinates, the bounding box’s aspect ratio $\rho_{f}$ is

(4) $$\rho_{f}=\frac{x_{\max}-x_{\min}}{y_{\max}-y_{\min}}$$

with $\rho_{f}>1$ indicating a prone or horizontal posture (possible fall) and $\rho_{f}<1$ indicating an upright posture. The prone states increment a fall counter with the requirement of 30 adjacent ones for fall confirmation (1 s continuity at 30 fps). This minimizes transient false alarms due to jitter or brief occlusions.

For fine-grained temporal analysis, we integrate a Transformer neural network to account for the sequential evolution of the landmarks. The Transformer model architecture includes the following:

- Input structure: The concatenated landmark coordinates as feature vectors of size 66 (33 points × 2 coordinates per point) for each of the 30 consecutive frames (1 s duration).
- Transformer Encoder: four stacked Transformer layers, each one having an 8-head self-attention mechanism to capture the complex spatiotemporal correlations and abrupt posture changes associated with falls.
- Classification Layer (Decoder): feedforward neural network to project the produced 256-dimensional embedding to a 1-dimensional output vector corresponding to “fallen” and “normal” classes via softmax probabilities.

The Transformer network is trained with manually labeled nighttime data, with 70% used for training, 15% for validation, and 15% for testing, Adam optimization (learning rate 3 × 10^−4^) with cross-entropy loss [39], and early stopping to avoid overfitting.

A secondary temporal verification step aggregates the bounding box and Transformer outputs to enforce a robust fall confirmation, thus significantly reducing the likelihood of false positives due to temporary landmark occlusions or lighting fluctuations.

### 3.2. LSTM Autoencoder for Inertial Abnormality Detection

The inertial sensing module consists of an unsupervised abnormality detection architecture based on an LSTM autoencoder. The model is as follows:

- Encoder: Two stacked LSTM layers, each one with 128 hidden units, to compress the inertial signals (tri-axial accelerations and gyroscopic velocities) into a compact 256-dimensional latent representation.
- Decoder: A symmetric LSTM-based decoder reconstructing the original inertial sequences from the latent vectors, followed by a dense output layer (256 to 6-dimensional reconstruction).

The model training involves predominantly normal inertial data (non-fall activities), segmented into sliding windows of 60 samples with 50% overlap. Z-score normalization and band-pass filtering are used to pre-process the IMU signals for drift and noise artifact reduction as will be described in Section 4.4. The hyperparameters were optimized with validation-based grid search [40].

The reconstruction error is quantified by the Mean Squared Error (MSE) at the autoencoder’s output:

(5) $$MSE\left(n\right)=\frac{1}{T}\;\sum_{t=1}^{T}{\left\|x_{n}^{t}-{\hat{x}}_{n}^{t}\right\|}^{2},\;T=60$$

where $x_{n}^{t}$ denotes the IMU input vector and ${\hat{x}}_{n}^{t}$ its corresponding reconstruction by the LSTM autoencoder. A statistical error threshold τ (e.g., 95th percentile) is used to detect abnormal motion patterns, with any reconstruction MSE exceeding τ triggering a motion abnormality alert for the associated window:

(6) $$Abnormal\;if\;MSE\left(n\right)>\;\tau \;$$

### 3.3. Decision-Level Fusion Rule

**Figure 2:**
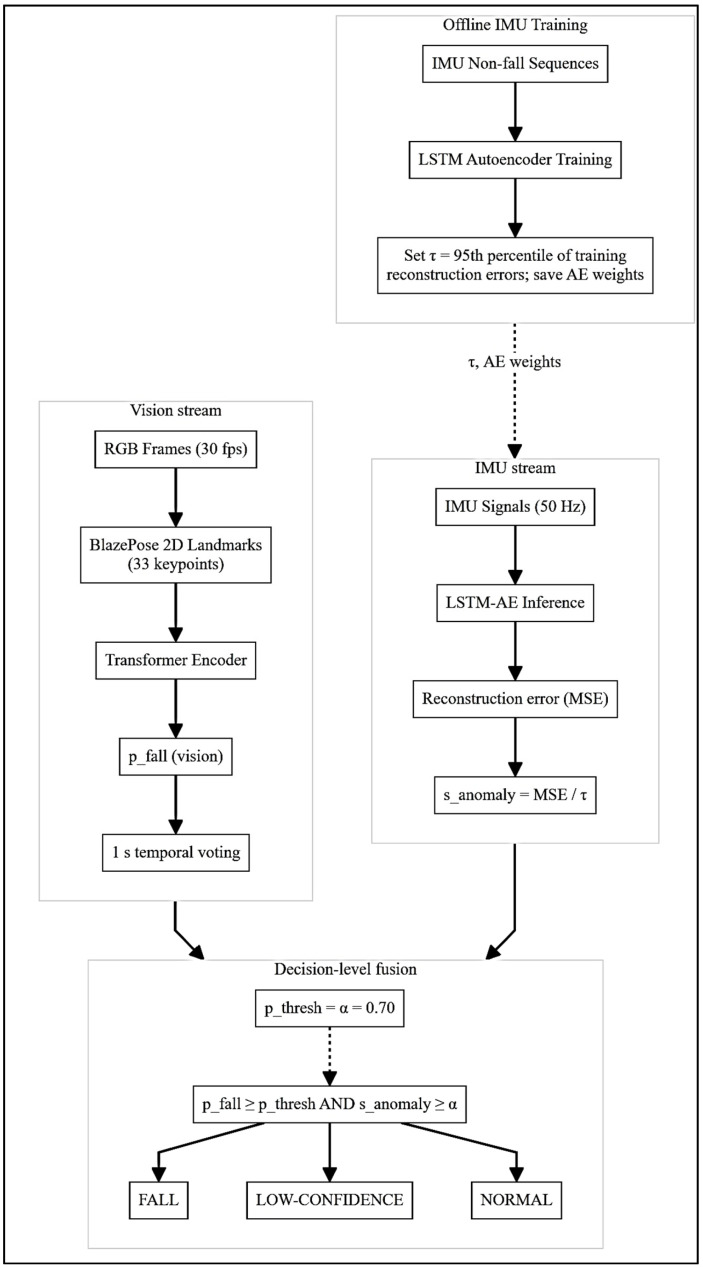
Proposed IMU–RGB pipeline with decision-level fusion for fall detection. Offline: the LSTM autoencoder is trained on non-fall IMU sequences and the 95th percentile of training reconstruction errors defines a threshold τ for detecting abnormal IMU sequences; the Transformer encoder is trained on vision data for binary FALL detection. *Runtime*: Vision stream: RGB video (30 fps) → BlazePose (33 2D landmarks) → Transformer inference → fall probability $p_{\mathrm{fall}}\;$ after 1 s temporal voting; IMU stream: IMU signal (50 Hz) → LSTM-AE inference → abnormality score $s_{\mathrm{anomaly}}=MSE/\tau$ after 1 s. Decision-level fusion: FALL if $p_{\mathrm{fall}}\;>p_{\mathrm{thresh}}\;$ and $s_{\mathrm{anomaly}}>\alpha$ ($p_{\mathrm{thresh}}\;=\alpha =0.70$ in this work); LOW-CONFIDENCE FALL if only one condition holds; NORMAL otherwise.

The final classification integrates the visual and inertial outputs through a decision-level fusion rule. Figure 2 depicts the runtime pipeline, showing the IMU and vision streams producing independent fall scores, each one compared to a common threshold *α* (0.70 used in this work), with a joint gating rule issuing the final label. More specifically, the late-fusion approach uses the vision-based fall probability $p_{fall}^{\left(Vision\right)}$ and the IMU-based abnormality score normalized into [0, 1]

(7) $$s_{\mathrm{anomaly}}=min\left(\frac{MSE(n)}{\tau },\;1\right)$$

A segment is labeled FALL when $p_{fall}^{\left(Vision\right)}>\alpha$ and $s_{\mathrm{anomaly}}>\alpha$ (with α=0.70). If only one score exceeds α, the event is flagged LOW-CONFIDENCE for further verification; otherwise, the label is NORMAL.

As mentioned in Introduction, this design improves robustness against single-modality failures. For example, false positives from inertial impulses (e.g., abrupt sitting) are suppressed by visual verification, whereas vision occlusions or darkness are compensated by reliable inertial detections.

### 3.4. Few-Shot Personalization Protocol

To evaluate the system’s adaptability to unseen users in data-scarce scenarios, we conducted a dedicated few-shot learning experiment atop the Leave-One-Subject-Out (LOSO) protocol, a variant of *k*-fold cross-validation [41] where each fold considers the data from a single subject. For each of the 16 participants, the model is first trained on the data from the remaining 15 subjects, yielding a subject-agnostic baseline. Subsequently, *K* annotated sequences from the held-out subject, $K\in \left\{1,2,\ldots ,10\right\},$ were used to incrementally fine-tune the final fusion classifier with the inertial and visual encoders frozen. This protocol emulates post-deployment calibration under limited supervision and transfer learning from the subject-agnostic model to a new user. Next is the detailed procedure:

Let $D=\left\{S_{1},S_{2},\ldots ,S_{16}\right\}$, where $S_{i}$ is a set of time-synchronized and recorded RGB and IMU sequences for subject $i\in \left\{1,\ldots ,16\right\},$ each sequence representing labeled data over a 1 s interval (30 RGB frames at 30 fps and 60 IMU samples at 50 Hz). The sampling is class-stratified to include both fall and non-fall segments when available. Then, the following three computations are performed for each value of *K*:

(1) LOSO pre-training: for every subject i, a baseline model M−i is trained on

(8) $$D_{train}^{\left(i\right)}=D\;\backslash \;S_{i}$$

(2) Incremental M−i fine-tuning with *K* sequences (few-shot). Using the held-out subject data, we uniformly sample without replacement a calibration set:

(9) $$D_{cal}^{\left(i,K\right)}\subset S_{i},\;\left|D_{cal}^{\left(i,K\right)}=K\right|,\;K\in \left\{1,2,\ldots ,10\right\}$$

where K is capped to 10 to model a realistic calibration effort and because performance saturates beyond K≈7 (see Section 5.5). As mentioned, only the decision-level fusion classifier is updated (learning rate 1 × 10^−4^, 50 iterations), while the IMU (LSTM) and vision (Transformer) encoders are frozen. As a result, the number of trainable parameters is less than 5 k, enabling on-device adaptation in less than 2 s on an average CPU such as Intel’s I5.

(3) Evaluation and aggregation: The adapted model $M_{i,K}$ is tested on the disjoint held out set

(10) $$D_{test}^{\left(i,K\right)}=S_{i}\setminus D_{cal}^{\left(i,K\right)}$$

For each K, we report the mean F1-score across 16 LOSO folds,

(11) $$\bar{F1(K)}=\frac{1}{16}\;\sum_{i=1}^{16}F1\left(M_{i,K},\;D_{test}^{\left(i,K\right)}\right),$$

and the 95% percent confidence intervals are computed across folds using Student’s *t* with 15 degrees of freedom:

(12) $$\bar{x}\pm \;t_{0.975,15}\;\frac{s}{\sqrt{16}}\;(with\;t_{0.975,15}\approx 2.131)$$

where $\bar{x}$ is the fold-wise mean, s is the sample standard deviation of the per-fold metric. The between-method differences are assessed with paired two-sided *t*-tests over the 16 folds (significance p < 0.05; here all p < 0.001), and the resulting $\bar{F1(K)}$ curve quantifies the few-shot recovery performance as a function of K.

## 4. Experimental Setup and Dataset Description

### 4.1. Participants and Experimental Setup

The experimental protocol was conducted with sixteen healthy adult volunteers (eight males, eight females; mean age: 42.1 ± 4.8 years; height: 1.71 ± 0.07 m; weight: 68.9 ± 9.4 kg), none of whom reported neurological, orthopedic, or cardiovascular conditions that might affect mobility. All participants provided written informed consent in accordance with institutional ethics guidelines prior to participation.

Data acquisition was carried out in a controlled indoor environment simulating a typical nocturnal bedroom scenario. The setup included a standard single bed, pillows, blankets, a bedside table, and other common furniture items to ensure ecological realism. Ambient illumination was maintained below 5 lux to replicate realistic nighttime conditions, without the use of auxiliary lighting or infrared sources.

### 4.2. Data Acquisition

**Figure 3:**
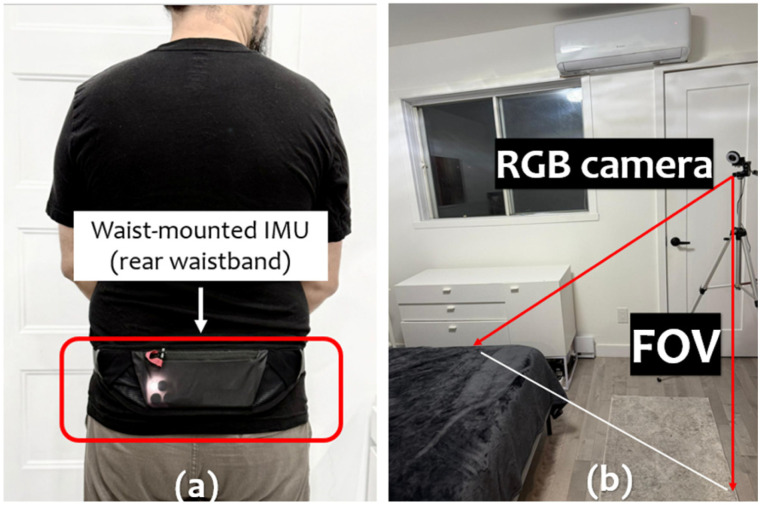
Experimental setup: (**a**) Waist-mounted IMU placed at the back (smartphone form factor). (**b**) RGB positioned at 1.2 m with an oblique lateral viewpoint, with the field of view (FOV) indicated in red and covering the bed area. Ambient illumination kept below 5 lux with no auxiliary IR lighting during data collection.

As Figure 3 shows, each subject wore a waist-mounted smartphone (rear waistband) secured using an adjustable elastic strap to ensure stable sensor contact. The embedded IMU (Inertial Measurement Unit) captured six-axis data: three-axis linear accelerations (±16 g) and three-axis angular velocities (±2000°/s), uniformly sampled at 50 Hz. All inertial data streams were timestamped and loosely synchronized with the RGB video stream using a local Network Time Protocol (NTP) server, achieving sufficient temporal coherence for decision-level fusion processing.

The RGB video data were recorded using a laterally positioned camera placed at approximately 1.2 m height to ensure a full and unobstructed view of each participant’s body during all activities. The video streams were acquired at 1920 × 1080 resolution and 30 fps. The room illumination was set below 5 lux for realistic nighttime conditions.

### 4.3. Experimental Protocol and Data Collection

Each participant completed a total of 30 scripted trials, comprising 15 simulated falls (covering forward, backward, and lateral directions) and 15 segments of routine nocturnal activities, including lying down, rolling over in bed, sitting up, standing from a lying position, and walking within the experimental environment. Each trial lasted approximately two minutes, yielding a total of roughly one hour of data per subject. For the entire cohort of 16 participants, this resulted in 16 h of multimodal recordings. To ensure ecological validity and preserve natural behavior, participants were instructed to execute each scenario with realistic motion patterns, without rigid constraints or robotic repetitions.

As mentioned in Section 3.2, the recorded inertial signals were segmented using a sliding window approach, with 60 samples per window corresponding to approximately 1.2 s, and 50% overlap between consecutive windows to preserve temporal continuity. The concomitant video recordings were divided into sequences of 30 consecutive frames per segment (corresponding to ~1 s at 30 fps), providing temporally aligned visual input for the vision-based model components.

Leave-One-Subject-Out (LOSO) cross-validation is employed to evaluate the model’s performance and to ensure robustness against inter-individual variability and to assess generalization to unseen subjects under realistic conditions.

### 4.4. Signal Processing and Feature Extraction

The raw IMU data underwent the following preprocessing steps:

- Z-score normalization to remove static offsets and standardize amplitude distributions across subjects.
- Filtering using a 4th-order Butterworth zero-phase digital filter with a band-pass frequency range from 0.2 Hz to 20 Hz, effectively eliminating low-frequency drift and high-frequency noise.

The resulting sequences were used as input for the IMU model in Section 3.2.

As already mentioned, the video frames were processed with MediaPipe BlazePose [38], providing real-time extraction of 33 anatomical landmarks with normalized coordinates (x, y), alongside visibility confidence scores ranging from 0 to 1. Landmarks with confidence below a threshold of 0.5 were discarded, ensuring robustness against occlusions and low illumination.

The landmarks’ bounding box was computed frame-by-frame to derive posture features as detailed in Section 3.1.

### 4.5. Deep Learning Models

The model’s architecture is described in Section 3.2 and training it was conducted on non-fall sequences, using mean squared error (MSE) loss and the Adam optimizer [39] (learning rate: 0.001, batch size: 32, 50 epochs, early stopping after 10 stagnant epochs).

The model’s architecture and temporal voting are described in Section 3.1. Training was supervised with manually annotated data, using cross-entropy loss optimized by Adam (learning rate 1 × 10^−4^, cosine annealing, batch size 64, 40 epochs, early stopping after 5 epochs without improvement).

### 4.6. Late Fusion Algorithm

The decision-level fusion rule (score computation, thresholds, and temporal vote) is formally defined in Section 3.3 and detailed in Supplementary Algorithm S3. For all experiments, a fixed threshold α=0.70 was used for both modalities, and a 1−s temporal vote was applied on the vision stream.

### 4.7. Evaluation Metrics

To thoroughly evaluate the system’s performance, standard metrics were used to provide a comprehensive insight into both positive event detection capability and false alarm suppression. They included the following:

$$Accuracy=\frac{TP+TN}{TP+TN+FP+FN}$$

$$Precision=\frac{TP}{TP+FP}$$

$$Recall\left(Sensitivity\right)=\frac{TP}{TP+FN}$$

$$Specificity=\frac{TN}{TN+FP}$$

$$F1\text{-}Score=2\times \frac{Precision\times Recall}{Precision+Recall}$$

### 4.8. Statistical Analysis

Comparative analyses between the proposed bimodal decision-level fusion model and the two single-modality baselines (IMU-only and vision-only) were conducted using paired-sample *t*-tests. A significance threshold of *p* < 0.05 was adopted to determine statistical relevance. Additionally, 95% confidence intervals were computed for the key performance metrics, precision, recall, and F1-score, to assess the statistical reliability and variability of the results across participants.

### 4.9. Computational Environment

All the deep learning models were implemented using PyTorch (v2.2.0; Meta Platforms Inc., Menlo Park, CA, USA). The experimental evaluations were conducted on a workstation equipped with an Intel Core i5-8265U processor (4 cores/8 threads, base 1.60 GHz, turbo up to 3.90 GHz), 8 GB of RAM, and no GPU to reflect deployment in relative resource-limited environments, hence providing an estimation of system performance for real-time applications in embedded or edge-based healthcare scenarios.

### 4.10. Sample-Size Adequacy via a Subject-Wise Learning Curve

To justify the cohort size, we computed a subject-wise learning curve under the LOSO protocol for sizes $m\;\in \left\{2,4,6,8,10,12,14,16\right\}$, with the models trained on randomly selected sets of m−1 training subjects and evaluated on the held-out subject, and the F1-scores averaged over all LOSO folds. Each m was repeated 10 times with different random draws, and we report the mean with 95% confidence intervals (Student’s t). We define saturation as a marginal gain <0.5 percentage points when increasing from m=12 to m=16. Then, the obtained leaning curve allows us to assess each cohort’s adequacy for training the system.

## 5. Results

**Figure 4:**
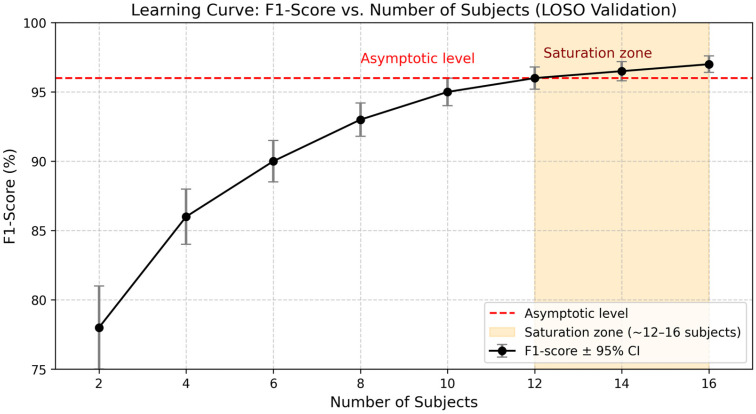
The red dashed line shows the estimated saturation level of the learning curve; error bars denote 95% confidence intervals. The shaded region (*n* ≥ 12) marks the plateau.

**Figure 5:**
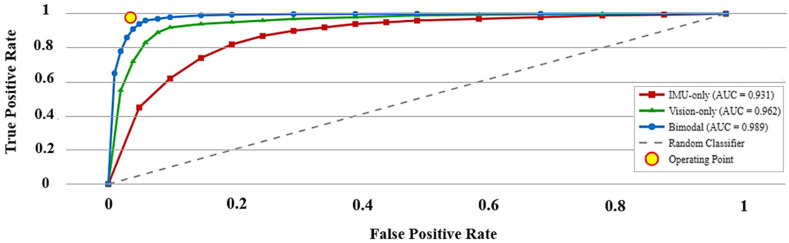
ROC Curves for the IMU-only LSTM auto-encoder, the vision-only Transformer, and the proposed bimodal late-fusion detector after 16 LOSO folds. The corresponding AUC means confirm the superior sensitivity–specificity trade-off of the fusion approach.

**Figure 6:**
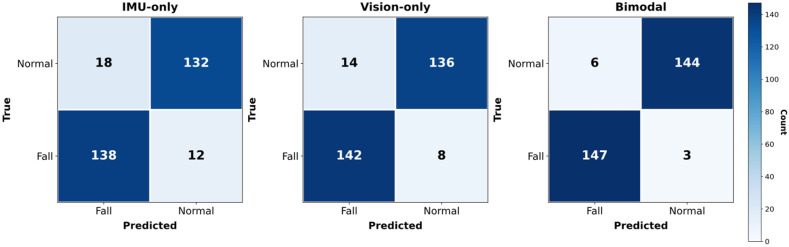
Typical confusion matrix over one LOSO fold (*N* = 16) for FALL versus NORMAL detections. Compared to the IMU baseline, the bimodal model reduces the false positive rate by 67% and the false negative rate by 75%, and compared to the vision baseline, those reductions are 57% and 63%, respectively.

The proposed IMU-RGB system was benchmarked against IMU-only and vision-only baselines under LOSO validation. The fusion model achieved 97.2% accuracy, 96.9% precision, 97.8% recall, 97.3% F1, 0.989 ± 0.012 AUC, and 3.6% FPR, with ≈50 ms per frame latency (~20 fps). Few-shot personalization recovered ≥95% of baseline performance with K = 5 labeled sequences. The detailed results are shown in Figure 4, Figure 5 and Figure 6 and Table 1. Latency and few-shot results are provided in Supplementary Materials.

### 5.1. Learning-Curve Analysis and Cohort Adequacy

Following the procedure in Section 4.10, the subject-wise learning curve in Figure 4 shows steep gains up to *N* = 10–12 participants before reaching a plateau at F1 = 96–97%. The red dashed line in Figure 4 marks the estimated asymptotic performance level $\hat{a}$, obtained by fitting a saturating exponential $F_{1}\left(n\right)=a-b\;e^{-cn}$ to the LOSO points (*n* = 2…16). The fitted asymptote is $\hat{a}=96.7\%$ (95% CI: 96.2–97.2%), and this result supports our choice of *N* = 16 for the present study, since the corresponding performance score of 97% is consistent with this which is consistent with the obtained plateau, providing enough headroom beyond the performance knee while keeping the study practical by avoiding further participant recruitment.

### 5.2. Global Performance Comparison

To establish a quantitative baseline, the proposed bimodal late-fusion model was systematically benchmarked against two unimodal configurations: (i) an IMU-only abnormality detector based on an LSTM autoencoder, and (ii) a vision-only classifier based on a Transformer architecture. All models were evaluated under identical conditions using LOSO cross-validation. Table 1 presents the average performance metrics computed across the 16 LOSO folds, where each subject served once as the held-out test case. For each metric, the reported values correspond to the mean and 95% confidence interval. The bimodal architecture consistently outperformed both unimodal baselines on all metrics, achieving an accuracy of 97.2%, precision of 96.9%, recall of 97.8%, specificity of 96.6%, and an F1-score of 97.3%. These gains were statistically significant (*p* < 0.001, paired *t*-test), demonstrating the synergistic effect of integrating inertial and visual modalities.

### 5.3. Receiver-Operating Characteristics, Error Structure, and False-Alarm Control

The discriminative capacity of the proposed system was assessed along three complementary dimensions: global ROC-based performance, class-specific error structure, and false-alarm suppression. These axes jointly characterize the model’s reliability under real-world deployment conditions. Figure 5 shows the obtained Receiver Operating Characteristic (ROC) curves computed across all LOSO validation folds. The proposed bimodal late-fusion system achieved an area under the curve (AUC) of 0.989 ± 0.012, markedly surpassing the vision-only Transformer (0.962 ± 0.015) and the IMU-only LSTM autoencoder (0.931 ± 0.018). These values confirm a superior trade-off between sensitivity and specificity for the bimodal system.

Confusion matrices were built to visualize the classification performance of the proposed late fusion bimodal model and two single-modal models used for comparison; Figure 6 shows an example from a single LOSO fold, leading to two key observations. First, the bimodal false positive rate is 67% lower than the IMU baseline and 57% lower than the vision baseline. Second, the false negative rate is concomitantly reduced by 75% and 63%, respectively. Using micro-averaging across all folds shows the global false-positive rate dropping from 11.3% (IMU) and 8.9% (vision) to only 3.6% under the proposed late fusion architecture. Such suppression of unnecessary alarms is critical for long-term, unobtrusive monitoring in domestic environments, where false alerts can undermine user confidence and compliance.

### 5.4. Computational Performance

A detailed latency decomposition is provided in the Supplementary Materials, including Table S1 and Figure S1.

### 5.5. Few-Shot Personalization Analysis

The subject-specific adaptation results are presented in Supplementary Materials (Figure S2), showing that ≥95% of baseline performance is recovered with only five labeled sequences.

## 6. Discussion

Under LOSO cross-validation with *N* = 16, the proposed CPU-only bimodal late-fusion framework achieved 97.2% accuracy, 97.8% recall, F1 = 97.3%, and FPR = 3.6%, while sustaining ≈20 fps (≈50 ms per frame). Requiring agreement between modalities consistently reduced false alarms relative to unimodal baselines without sacrificing sensitivity—essential for long-term acceptability in home monitoring. The learning-curve analysis (Section 5.1) shows performance gains saturate at F1 ≈ 96–97% beyond ≈12 subjects, supporting the adequacy of *N* = 16 for this pilot. Latency is dominated by pose-landmark extraction; Transformer/LSTM inference overheads are small and decision-level fusion is negligible, confirming feasibility for edge deployment under nocturnal conditions.

### 6.1. Benefits of Decision-Level Fusion over Unimodal and Early-Fusion Approaches

The IMU stream (LSTM autoencoder) is sensitive to sharp accelerometric impulses but can misclassify abrupt yet benign transitions (e.g., rapid sitting), whereas the vision stream (Transformer over 2D landmarks) captures postural context but degrades under occlusion and low light. Processing streams independently and fusing at the decision stage prevents the noise propagation typical of early fusion, reducing FPR from 11.3% (IMU-only) and 8.9% (vision only) to 3.6% while maintaining high recall (97.8%) and AUC = 0.989 ± 0.012 (Section 5.2). Under dim lighting, Li et al. [5] report 90.2% accuracy with a 12% FPR, while Feng et al. [34] achieve <2% FPR in controlled labs using depth cameras; our approach attains 97.2% accuracy with a 3.6% FPR in realistic nocturnal scenes using commodity RGB+IMU, narrowing the gap without specialized hardware.

### 6.2. Real-Time Execution and Latency Profile

On a modest CPU platform such as one using the Intel Core i5-8265U, the end-to-end inference time is ≈50.0 ± 4.7 ms per frame. The pose-landmark extraction accounts for ≈62% of the runtime, while the Transformer and LSTM inferences are minor contributors, and the late fusion process adds a negligible cost. If additional speed is required, optimization should prioritize the landmark extraction (input down-sampling, quantization, or lighter keypoint backbones) rather than the fusion rule. On another front, and compared with IR/depth solutions, the commodity RGB hardware typically reduces device cost by ≈5–10× and avoids multi-sensor calibration, while our pipeline sustains AUC = 0.989 below 5 lux illumination.

### 6.3. Few-Shot Learning Capabilities and Personalization

In practical deployments, all the operating points are data-driven rather than hand-tuned. The IMU abnormality gate τ is initialized as the 95th percentile of training reconstruction errors, while the vision decision threshold and the fusion gate (both 0.70) are selected via ROC analysis on validation folds. At installation, an automatic calibration routine (i) records a brief baseline of normal activity (5–10 min) to update τ for the device, and (ii) optionally performs few-shot personalization (≤5 short labeled sequences) to adapt the final fusion layer to the user and site. This procedure removes per-dataset manual tuning and yields stable operating points across environments.

### 6.4. Communication and Alerting Pipeline (Deployment Guidance)

For real-world deployment, a reliable communication layer is required to complement the proposed on-device fall detection framework. To this end, we specify a minimal, standards-based pipeline designed for home-care integration. In this architecture, the fall detection remains fully processed locally on the device to ensure privacy, and only the event metadata is transmitted externally, with the detections conveyed as authenticated MQTT/HTTPS messages including timestamp, confidence, and modality flags. The communication stack supports acknowledgment, retry with local buffering during temporary outages, and configurable escalation channels (e.g., SMS or automated voice calls) for high-confidence events. End-to-end alert latency is targeted at below 2 s, which is consistent with healthcare monitoring requirements. To guarantee auditability and maintain IMU–camera alignment, network time synchronization (e.g., NTP) is enforced.

This communication layer thus represents a practical requirement for deployment, allowing the seamless integration into assisted-living infrastructures and providing a foundation for future large-scale validation studies.

### 6.5. Population Considerations: Elderly Biomechanics

Elderly falls often exhibit lower peak accelerations, slower descent or seated-collapse patterns, and kyphotic posture. These traits can damp IMU impulses and shift pose dynamics. Accordingly, the system: (a) lengthens the temporal vote when slow descent is detected, (b) slightly relaxes the IMU abnormality gate under sustained low-energy deviations, and (c) prioritizes few-shot personalization to capture user-specific kinematics. A follow-up study with older adults will quantify these adaptations.

### 6.6. Deployment Outlook and Cost-Efficient Scalability

The data processing is performed on a device with a default skeleton-only retention policy (2D keypoints) with zero storage of RGB frames. If the frames must be retained (e.g., for audit), face/body blurring, encryption in transit and at rest, role-based access, and short retention with explicit consent must be applied. Moreover, the camera placement uses oblique viewpoints to reduce identifiability. In any case, the privacy settings can be configurable for consistency with application site policy.

### 6.7. Limitations and Future Work

This study involved healthy adults (30–50 s) and simulated falls under controlled nocturnal conditions with a single lateral RGB camera and waist IMU. Real-world clutter, multi-person scenes, and true elderly falls may introduce additional variance. Future work will expand to older cohorts and longitudinal deployments, explore on-device adaptive thresholds, evaluate 3D/IR variants where lighting permits, and integrate ambient sensors (e.g., pressure mats) for further false-alarm suppression.

## 7. Conclusions

The proposed decision-level IMU-RGB framework achieves 97.2% accuracy, 97.8% recall, and a 3.6% false-positive rate at ~20 fps on CPU-only hardware, indicating practical readiness for nighttime home monitoring. Automatic calibration and few-shot personalization remove manual tuning and adapt to user variability, supporting real-world deployment with on-device processing and configurable privacy safeguards.

## Figures and Tables

**Table 1:** Mean performance (±95% confidence intervals) under LOSO validation (*N* = 16). The metrics are computed per fold before averaging, with the best values shown in bold. Paired two-sided *t*-tests (Bimodal vs. IMU-only and Bimodal vs. Vision-only) are significant for all metrics (*p* < 0.001).

Method	Accuracy (%)	Precision (%)	Recall (%)	Specificity (%)	F1-Score (%)	FPR (%) ^1^
IMU-only (LSTM)	90.3 ± 1.1%	89.6 ± 1.2%	91.7 ± 1.0%	88.7 ± 1.4%	90.6 ± 1.1%	11.3 ± 1.4%
Vision-only (Transformer)	92.9 ± 0.9%	92.2 ± 1.0%	94.0 ± 0.8%	91.1 ± 1.1%	93.1 ± 0.9%	8.9 ± 1.2%
Bimodal Late Fusion	**97.2 ± 0.6%**	**96.9 ± 0.6%**	**97.8 ± 0.5%**	**96.6 ± 0.7%**	**97.3 ± 0.6%**	**3.6 ± 0.6%**

^1^ FPR = False Positive Rate.

## Data Availability

The original contributions presented in this study are included in the article/Supplementary Materials. Further inquiries can be directed to the corresponding authors.

## Supplementary Materials

The following supporting information can be downloaded at: https://www.mdpi.com/article/10.3390/s25196035/s1.

## References

[1] Wang X., Ellul J., Azzopardi G. (2020). Elderly Fall Detection Systems: A Literature Survey. Front. Robot. AI.

[2] Iguchi Y., Lee J.H., Okamoto S. Enhancement of Fall Detection Algorithm Using Convolutional Autoencoder and Personalized Threshold. Proceedings of the Digest of Technical Papers—IEEE International Conference on Consumer Electronics.

[3] Figueiredo I.N., Leal C., Pinto L., Bolito J., Lemos A. (2016). Exploring Smartphone Sensors for Fall Detection. mUX J. Mob. User Exp..

[4] Wang C.T., Liu Z., Chen K.H. (2016). A Wearable Accelerometer System for Fall Detection in the Elderly. IEEE Trans. Biomed. Eng..

[5] Li X.Y., Zhao W., Wang R. Vision-Based Fall Detection in Low-Light Environments. Proceedings of the IEEE ICCV Workshops.

[6] Thompson L.Z., Lee E.K., Huang C.M. (2016). SVM Classification of Accelerometer Data for Fall Detection. Eng. Appl. Artif. Intell..

[7] Malekzadeh M., Clegg R.G., Cavallaro A., Haddadi H. Mobile Sensor Data Anonymization. Proceedings of the 2019 IEEE International Conference on Pervasive Computing and Communications (PerCom).

[8] Vaswani A., Shazeer N., Parmar N., Uszkoreit J., Jones L., Gomez A.N., Kaiser L., Polosukhin I., Guyon I., Luxburg U.V., Bengio S., Wallach H., Fergus R., Vishwanathan S., Garnett R. (2017). Attention Is All You Need. Proceedings of the Advances in Neural Information Processing Systems 30 (NeurIPS 2017).

[9] Wang Y., Yao Q., Kwok J.T., Ni L.M. (2020). Generalizing from a Few Examples: A Survey on Few-Shot Learning. ACM Comput. Surv..

[10] Fang H.A., Tsai T., Pal A. (2019). Pose-Based Fall Detection via Skeleton Analysis. Pattern Recognit. Lett..

[11] Bian Z.-P., Hou J., Chau L.-P., Magnenat Thalmann N. (2015). Fall Detection Based on Body Part Tracking Using a Depth Camera. IEEE J. Biomed. Health Inform..

[12] Roberts M.C., Varghese P., Guo L. (2019). Privacy-Preserving Video Monitoring for Assisted Living. J. Ambient. Intell. Smart Environ..

[13] Hochreiter S., Schmidhuber J. (1997). Long Short-Term Memory. Neural Comput..

[14] Yu X., Feng Z., Wang H., Tang Y., Zhang Y. (2024). A Novel Semi-Supervised Model for Pre-Impact Fall Detection with Limited Fall Data. Eng. Appl. Artif. Intell..

[15] Kepski M., Kwolek B. Fall Detection Using Ceiling-Mounted 3D Depth Camera. Proceedings of the VISAPP 2014—Proceedings of the 9th International Conference on Computer Vision Theory and Applications.

[16] Zambanini S., Machajdik J., Kampel M. (2010). Early versus Late Fusion in a Multiple Camera Network for Fall Detection. Proceedings of the Workshop of the Austrian Association for Pattern Recognition.

[17] Baltrusaitis T., Ahuja C., Morency L.P. (2019). Multimodal Machine Learning: A Survey and Taxonomy. IEEE Trans. Pattern Anal. Mach. Intell..

[18] Lachekhab F., Benzaoui M., Tadjer S.A., Bensmaine A. (2024). LSTM-Autoencoder Deep Learning Model for Abnormality Detection in Electric Motor. Energies.

[19] Kibet D., Muthee R., Wafula C., Muriuki G. (2024). Sudden Fall Detection of Human Body Using Transformer Model. Sensors.

[20] Lee J.-S., Tseng H.-H. (2019). Enhanced Threshold-Based Fall Detection Using Smartphones. IEEE Sens. J..

[21] Cai W., Qiu L., Li W., Yu J., Wang L. Practical Fall Detection Algorithm Based on AdaBoost. Proceedings of the Proceedings of the ACM International Conference on Biomedical Signal and Image Processing.

[22] Lai C.-F., Chang S.-Y., Chao H.-C. (2011). Detection of Cognitive Injured Body Region Using Multiple Triaxial Accelerometers for Elderly Falling. IEEE Sens. J..

[23] Balli S., Sagbas E.A., Peker M. (2019). Human Activity Recognition from Smart Watch Sensor Data Using PCA and Random Forest. Meas. Control.

[24] Hakim A., Huq M.S., Shanta S., Ibrahim B.S. (2017). Smartphone-Based Data Mining for Fall Detection. Procedia Comput. Sci..

[25] Zhang J., Li Z., Liu Y., Li J., Qiu H., Li M., Hou G., Zhou Z. (2024). An Effective Deep Learning Framework for Fall Detection: Model Development and Study Design. J. Med. Internet Res..

[26] Espinosa R., Ponce H., Gutiérrez S., Martínez-Villaseñor L., Brieva J., Moya-Albor E. (2019). A vision-based approach for fall detection using multiple cameras and convolutional neural networks: A case study using the UP-Fall detection dataset. Comput. Methods Programs Biomed..

[27] Rafferty J., Synnott J., Nugent C., Morrison G., Tamburini E. (2016). Fall Detection Through Thermal Vision Sensing.

[28] Willems J., Debard G., Vanrumste B., Goedemé T. (2009). A Video-Based Algorithm for Elderly Fall Detection.

[29] Kwolek B., Kepski M. (2014). Human Fall Detection on Embedded Platform Using Depth Maps and Wireless Accelerometer. Comput. Methods Programs Biomed..

[30] Martínez-Villaseñor L., Ponce H., Brieva J., Moya-Albor E., Núñez-Martínez J., Peñafort-Asturiano C. (2019). Up-Fall Detection Dataset: A Multimodal Approach. Sensors.

[31] Ozcan K., Velipasalar S., Varshney P.K. (2016). Autonomous Fall Detection with Wearable Cameras by Using Relative Entropy Distance Measure. IEEE Trans. Hum. Mach. Syst..

[32] Castillo J.C., Carneiro D., Serrano-Cuerda J., Novais P., Fernández-Caballero A., Neves J. (2014). A Multi-Modal Approach for Activity Classification and Fall Detection. Int. J. Syst. Sci..

[33] Shin J., Miah A.S.M., Egawa R., Hassan N., Hirooka K., Tomioka Y. (2025). Multimodal Fall Detection Using Spatial-Temporal Attention and Bi-LSTM-Based Feature Fusion. Future Internet.

[34] Feng P., Yu M., Naqvi S.M., Chambers J.A. Deep Learning for Posture Analysis in Fall Detection. Proceedings of the Proceedings of the IEEE Digital Signal Processing Conference.

[35] Cash J.J. (2009). Alert Fatigue. Am. J. Health-Syst. Pharm..

[36] Liu C.-L., Lee C.-H., Lin P.-M. (2010). A Fall Detection System Using K-Nearest Neighbor Classifier. Expert. Syst. Appl..

[37] Lugaresi C., Tang J., Nash H., McClanahan C., Uboweja E., Hays M., Zhang F., Chang C.-L., Yong M.G., Lee J. MediaPipe: A Framework for Perceiving and Processing Reality. Proceedings of the IEEE CVPR Workshops.

[38] Bazarevsky P., Kartynnik Y., Grishchenko I., Grundmann M. BlazePose: On-Device Real-Time Body Pose Tracking. https://arxiv.org/abs/2006.10204.

[39] Kingma D.P., Ba J.L. Adam: A Method for Stochastic Optimization. Proceedings of the 3rd International Conference on Learning Representations.

[40] Bergstra J., Bengio Y. (2012). Random Search for Hyper-Parameter Optimization. J. Mach. Learn. Res..

[41] Hastie T., Tibshirani R., Friedman J. (2009). The Elements of Statistical Learning.

[42] Google AI for Developers (2025). Pose Landmark Detection Guide—MediaPipe Documentation. https://ai.google.dev/edge/mediapipe/solutions/vision/pose_landmarker#pose_landmarker_model.

