# Supplementary material for: Fall Detection by Deep Learning-Based Bimodal Movement and Pose Sensing with Late Fusion
Source: Sensors (Basel). 2025 Oct 1;25(19):6035. doi: 10.3390/s25196035 (PMC12526565; doi:10.3390/s25196035)
Supplement: Supplementary file 1 [file sensors-25-06035-s001.zip › sensors-3854638-supplementary.pdf]

sensors

# Supplementary Materials

## Contents


- Algorithm S1: Vision-based temporal voting
- Algorithm S2: IMU anomaly detection with LSTM autoencoder
- Algorithm S3: Decision-level fusion rule
- Algorithm S4: Inference pipeline
- Table S1: Latency decomposition
- Figure S1: Latency profile and execution breakdown
- Figure S2: Subject-specific adaptation results

## Algorithm S1. Vision-based temporal voting

```
Call: TrainLSTMAutoencoder ( X_train, X_val,
                             h_dim = 128, T = 60, in_features = 6,
                             lr = 1e-3, batch_size = 32,
                                 max_epochs, patience, tolerance) → model
Input: X_train, X_val
Output: model

Body:
# ──────────Model creation and training──────────
model     ← buildLSTMAE(h_dim, T, in_features)
optimizer ← Adam(lr)

  for epoch in 1..max_epochs:
    train_loss = 0
    for batch in iterateBatches(X_train, batch_size = 32):
      recon = model.forward(batch)
      loss = MSE(recon, batch)
      optimizer.zeroGrad()
      loss.backward()
      optimizer.step()
      train_loss += loss.value

    val_loss = evaluateMSE(model, X_val)
    if val_loss > best_val_loss − tolerance:
      no_improvement_count += 1
      if no_improvement_count >= patience:
        break
    else:
      best_val_loss = val_loss
      no_improvement_count = 0
  return model
```

## Algorithm S2. IMU anomaly detection with LSTM autoencoder

$Call$: $VisionFallClassifier(frames = \{f1 \dots fK\},\quad p\_thresh = 0.70,$
$T = 30, L = 4, heads = 8,\quad d\_model = 256,$
$vis\_threshold = 0.5, shift = 15)$
$\rightarrow \{p_k(Vision),\quad lying\_bbox\}$

$Input$: $video\ frames\ \{f1 \dots fK\}\ (30\ fps)$; $p\_thresh \leftarrow 0.70$
$Output$: $(i)\ per - window\ fall\ probabilities\ p_k(Vision)$
$(ii)\ per - window\ lying\_bbox\ flag$

$Body$ :

\# ———— $Pose\ extraction\ (MediaPipe)$ ————

$pose \leftarrow BlazePose$ ($\text{def}_{conf} = 0.8, track_{conf} = 0.8$) → landmark detector
$buf \leftarrow RingBuffer(T = 30)$ → 30 frames ≈ 1 s

\# ———— $Temporal\ modelling\ (Transformer)$ ————

$trans \leftarrow VisionTransformer(L = 4, heads = 8, d_{model} = 256)$
$for\ each\ incoming\ frame\ ft\ do$
$\#\ MediaPipe: pixel \rightarrow 33\ landmarks$
$img \leftarrow BGR2RGB(ft)$
$res \leftarrow pose.process(img)$ → 33 landmarks + visibility

\# ———— Geometric cue (per − frame) ————

$w \leftarrow bbox\_width(res)$ ; $h \leftarrow bbox\_height(res)$
$lying\_bbox \leftarrow (w > h) \leftarrow aspect - ratio\ test$

\# ———— Semantic cue (per − window) ————

$coords \leftarrow landmarkTensor(res, vis_{threshold} = 0.5) \leftarrow 66 - D$
$buf.push(coords)$

$if\ buf.is\_full()\ then$
$X \leftarrow buf.tensor() \rightarrow shape\ (30, 66)$
$logits \leftarrow trans(X) \rightarrow Transformer$
$p\_fall \leftarrow softmax(logits)[Fall]$
$p_k(Vision) \leftarrow p\_fall$
$buf.slide(shift = 15) \rightarrow 50\ \%\ overlap$
$end\ if$
$end\ for$

## Algorithm S3. Decision-level fusion rule

$Call$: $VisualVote(lying\_bbox\_t, lying\_trans\_t, \tau\_frames = 30) \rightarrow vote\_t$
$\# \tau\_frames = 30 \approx 1\, s\ at\ 30\, fps$

$Input:$ $lying\_bbox\_t \in \{0,1\}$ $\# from\ Algorithm\ 2$
$lying\_trans\_t \in \{0,1\}$ $\#\ 1\ \ iff\ \ (p_k(Vision) > p_{thres})$
$State: c \in \mathbb{N}$ $\#\ persistent\ global\ counter\ (init\ 0)$
$Output: vote\_t \in \{0,1\}$ $\#\ 1 = confirm\ visual\ fall\ for\ this\ window$

$Body:$
$\#$ ———————————— $Vote$ ————————————
$if\ (lying\_bbox = 1)\ or\ (lying\_trans = 1)\ then$
$c \leftarrow c + 1$
$else$
$c \leftarrow 0$
$end\ if$
$if\ c \geq \tau\_frames\ then$
$vote\_t \leftarrow 1$ $\#\ confirm\ fall$
$c \leftarrow 0$
$else$
$vote\_t \leftarrow 0$

$end\ if$

## Algorithm S4. Inference pipeline

$Call$: $LateFusionDecision(p_{fall}^{(Vision)}, s_{abnormal}; p_{thresh} = 0.70, \alpha = 0.70) \rightarrow label\_t$

Input : $p_{fall}^{(Vision)} \in [0,1] \rightarrow$ Fall probability from Vision stream
$s_{abnormal} \in [0,1] \rightarrow$ Abnormality score from LSTM-AE
$p_{thresh} = 0.70 \rightarrow$ Vision confidence threshold
$\alpha = 0.7 \rightarrow$ IMU confidence threshold

Output : $label_t \in$ {Fall, Low-Confidence, Normal}

$Body$

\# ——— $Fused\ decision$ ———

if $(p_{fall}^{(Vision)} > p_{thresh}) \wedge (s_{abnormal} > \alpha)$ then
    $label_t \leftarrow Fall$ (High visual and inertial evidence)
elseif $(p_{fall}^{(Vision)} > p_{thresh}) \veebar (s_{abnormal} > \alpha)$ then
    $label_t \leftarrow$ Low-Confidence $\rightarrow$ only one modality is confident
else
    $label_t \leftarrow$ Normal $\rightarrow$ no modality provides strong evidence
end if
$return\ \ label_t$

## Table S1. Latency decomposition

| Component | Mean latency (ms)[1] | % of total computation |
|---|---|---|
| IMU preprocessing | 6.1 ± 1.0 | 12.2% |
| Vision landmark extraction | 31.2 ± 3.8 | 62.4% |
| LSTM inference | 4.7 ± 0.8 | 9.4% |
| Transformer inference | 5.3 ± 0.9 | 10.6% |
| Late fusion and overhead | 2.7 ± 0.4 | 5.4% |
| Total latency | 50.0 ± 4.7 | 100% |

[1] Latencies reported as mean ± standard deviation.

The real-time feasibility of the proposed bimodal architecture was quantified under CPU-only conditions (Intel Core i5-8265U, 8 GB RAM; no GPU). Table S1 reports per-module execution times and percentage loads; Figure S1 visualizes both absolute (ms) and relative (%) breakdowns. Vision-based landmark extraction dominates the budget (~62%), while the full pipeline remains at 50.0 ± 4.7 ms per frame (~20 fps), satisfying real-time constraints.

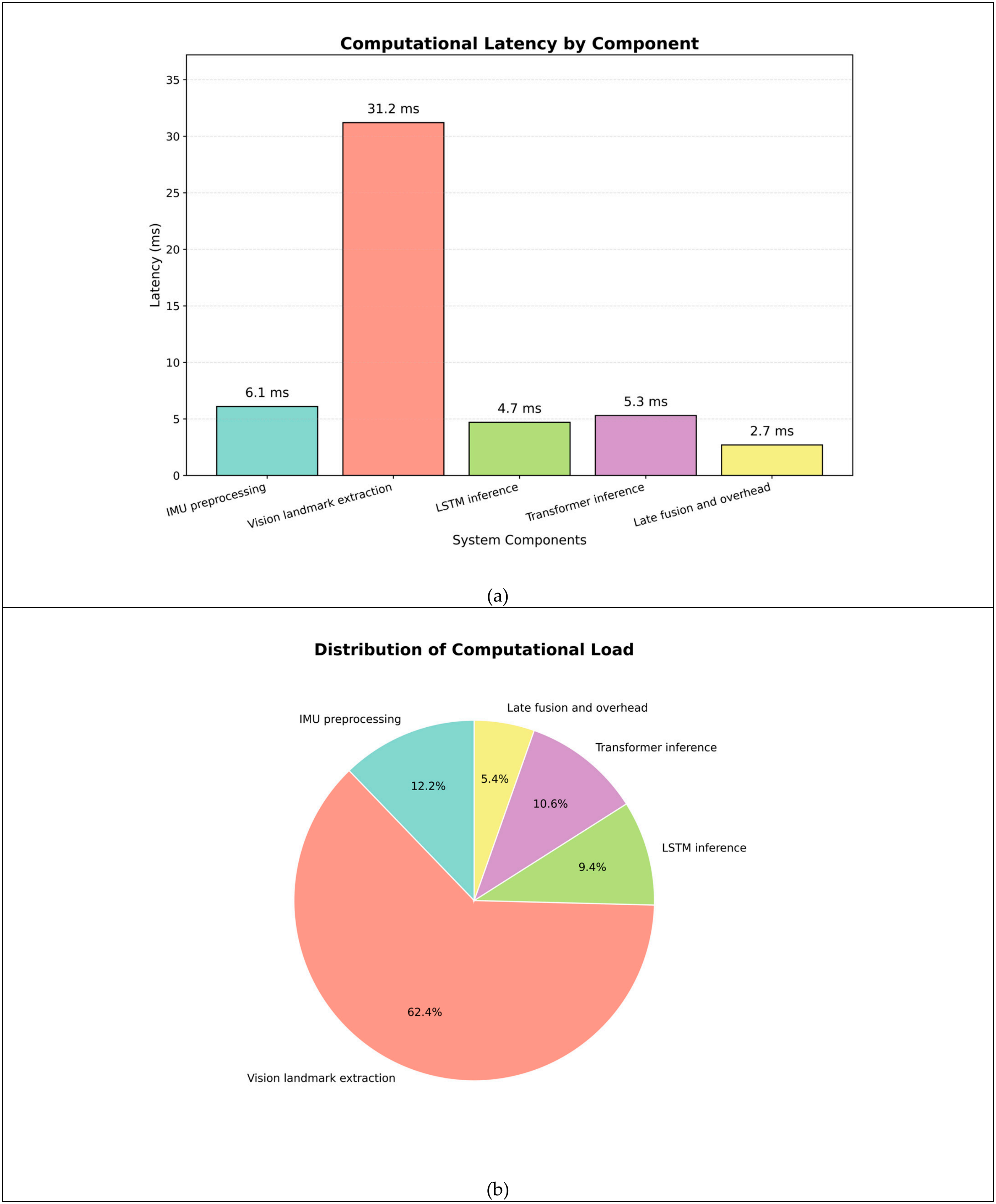


**Figure S1.** Latency profile and execution breakdown under CPU-only conditions (Intel Core i5-8265U, 8 GB RAM; no GPU). (a) Absolute per-module latencies (mean ± SD, ms). (b) Percentage contribution of each module to total computation. The pipeline totals 50.0 ± 4.7 ms per frame (~20 fps), with vision landmark extraction contributing ~62% of the budget.

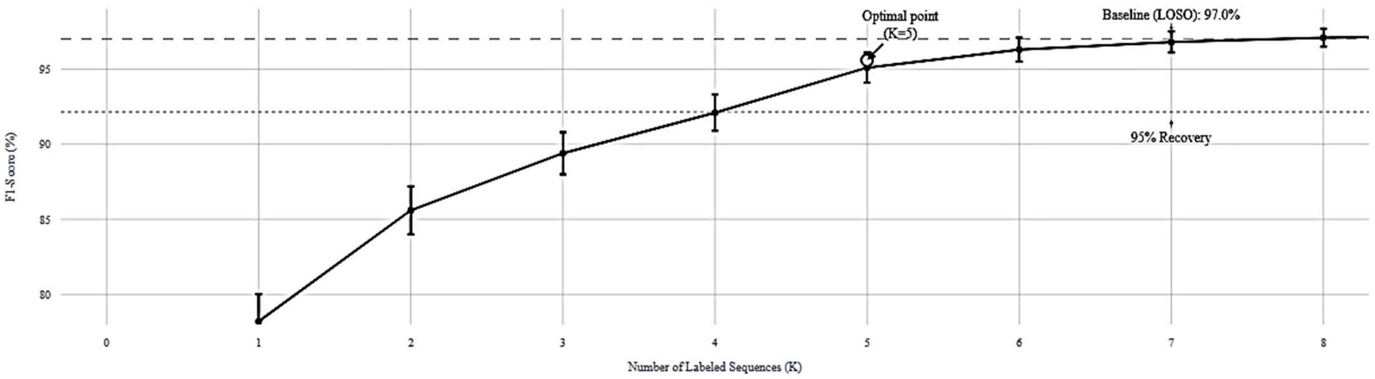


**Figure S2.** Few-shot learning performance recovery (LOSO, N = 16). Mean F1-score (±95% CI) as a function of the number of labeled sequences $K$ used for subject-specific fine-tuning. The dashed lines indicate the 95% recovery threshold and the full LOSO baseline (97.0%). The model achieves 95% of its full performance with only $K = 5$ annotated samples and saturates for $K \geq 7$.
